# Supplementary figures and images for: Validation study of Boil & Spin Malachite Green Loop Mediated Isothermal Amplification (B&S MG-LAMP) versus microscopy for malaria detection in the Peruvian Amazon
Source: PLoS One. 2021 Oct 25;16(10):e0258722. doi: 10.1371/journal.pone.0258722 (PMC8544869; doi:10.1371/journal.pone.0258722)

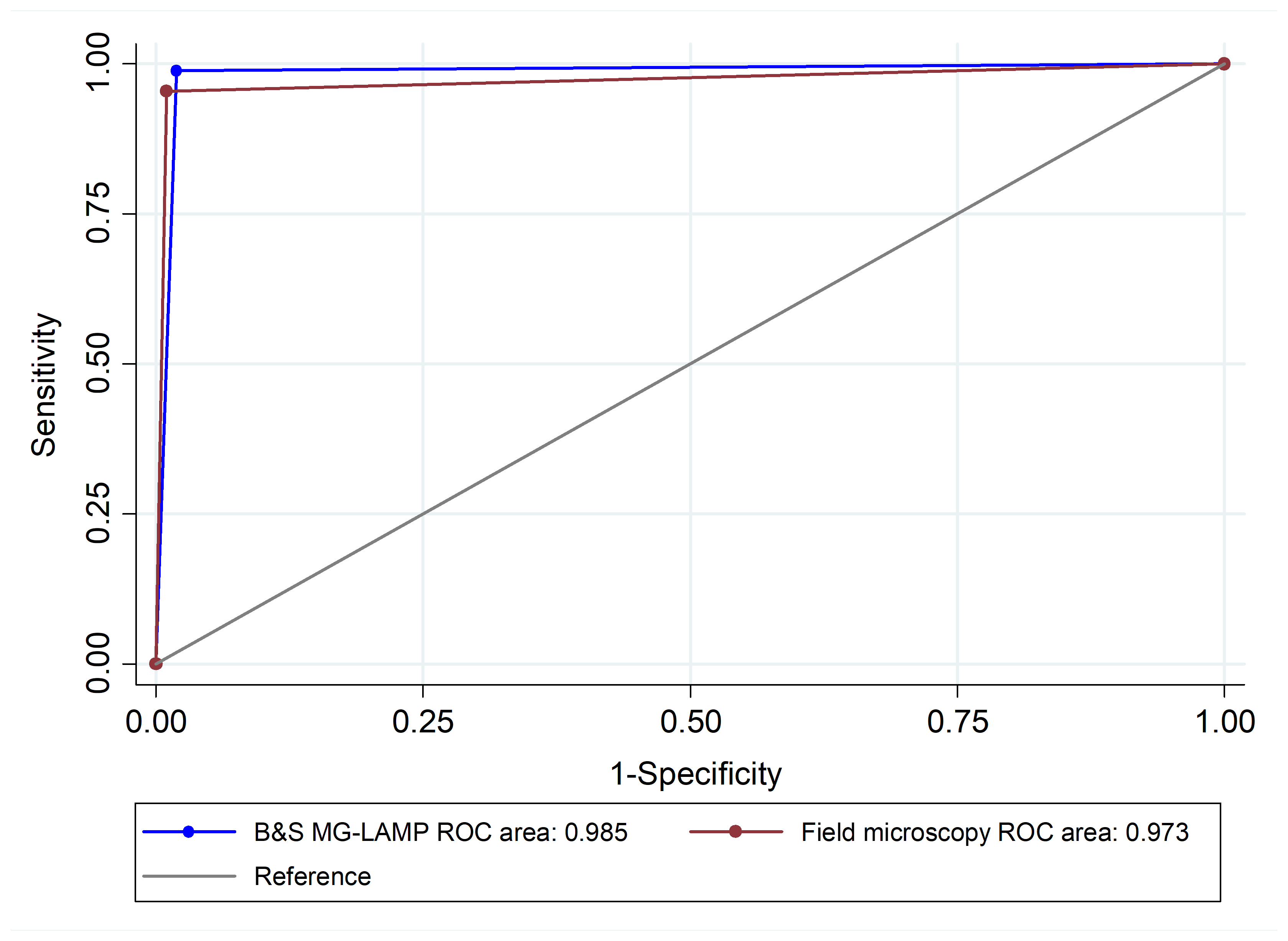

Supplement: S1 Fig — (TIF) [file pone.0258722.s001.tif]
