# Supplementary material for: Validation study of Boil & Spin Malachite Green Loop Mediated Isothermal Amplification (B&S MG-LAMP) versus microscopy for malaria detection in the Peruvian Amazon
Source: PLoS One. 2021 Oct 25;16(10):e0258722. doi: 10.1371/journal.pone.0258722 (PMC8544869; doi:10.1371/journal.pone.0258722)
Supplement: S1 Table — (DOCX) [file pone.0258722.s003.docx]

|  | *Plasmodium*spp | *P. falciparum* | *P. vivax* |
| --- | --- | --- | --- |
| Target | **18sr DNA gene** | **18sr DNA gene** | **R64 gene** |
| FIP | GGT GGA ACA CAT TGT TTC ATT TGA TCT CAT TCC AAT GGA ACC TTG | CAC CTA GTC GGT ATA GTT TAT GGT GCC TAA TCT ATT TCC ATT AAT | ATA TGG TCT CTC GAC ACG GCC AAA TTG CCA TCA TCT TCA C |
| BIP | GTT TGC TTC TAA CAT TCC ACT TGC CCG TTT TGA CCG GTC ATT | GTA GCA TTT CTT AGG GAA TGT TGG CCC CAG AAC CCA AAG ACT TTG A | TGT GCC CAC CCA CAT ACT TGG GGA AAT GTT AAT GGG GAT GT |
| F3 | TCG CTT CTA ACG GTG AAC | GAG GTG AAA TTC TAA GAT TTT CT | TCT GTT GGT GGA GTA GAT CC |
| B3 | AAT TGA TAG TAT GAG CTA TCC ATA G | TTC CGT CAA TTC TTT TAA CTT TC | CCT ACG TTT TGG TGA ATC G |
| LPF | CAC TAT ACC TTA CCA ATC TAT TTG AAC TTG | GGT ATC TGA TCG TCT TCA CTC CC | AGG CTA CTT CTT TTG CTC C |
| LPB | TGG ACG TAA CCT CCA GGC | GAA TTG CTT CCT TCA GTA CCT TA | ACT TAC AGT GCT GTA GAG A |
