# Supplementary material for: Validation study of Boil & Spin Malachite Green Loop Mediated Isothermal Amplification (B&S MG-LAMP) versus microscopy for malaria detection in the Peruvian Amazon
Source: PLoS One. 2021 Oct 25;16(10):e0258722. doi: 10.1371/journal.pone.0258722 (PMC8544869; doi:10.1371/journal.pone.0258722)
Supplement: S3 Table — (DOCX) [file pone.0258722.s005.docx]

| Method | Assay type | Sensitivity %, (95%CI) | Specificity %, (95%CI) |
| --- | --- | --- | --- |
| B&S MG-LAMP | genus | 99.4% (96.9 – 100%) | 97.1% (91.9 – 99.4%) |
|  | *P.falciparum* | 100% (78.2 – 100%) | 99.3% (97.3 – 99.9%) |
|  | *P.vivax* | 99.4% (96.6 – 100%) | 99.2% (95.5 – 100%) |
| Field Microscopy | genus | 97.2% (93.6 – 99.1%) | 99% (94.8 – 100%) |
|  | *P.falciparum* | 93.3% ( 68.1 – 99.8%) | 98.5% (96.2 – 99.6%) |
|  | *P.vivax* | 96.3% (92.1 – 98.6%) | 100% (97 – 100%) |
