# Supplementary material for: Validation study of Boil & Spin Malachite Green Loop Mediated Isothermal Amplification (B&S MG-LAMP) versus microscopy for malaria detection in the Peruvian Amazon
Source: PLoS One. 2021 Oct 25;16(10):e0258722. doi: 10.1371/journal.pone.0258722 (PMC8544869; doi:10.1371/journal.pone.0258722)
Supplement: S4 Table — Comparison of results of mixed infections (Pf/Pv) performed by each methodology. (DOCX) [file pone.0258722.s006.docx]

| **Samples** | **Field Microscopy** | **Expert Microscopy** | **B&S MG-LAMP** | **PET-PCR** | | |
| --- | --- | --- | --- | --- | --- | --- |
|  |  |  |  | **Species result** | **parasites/uL for Pf** | **parasites/uL for Pv** |
| 1 | Pf | Pf/Pv | Pf | Pf | 786.3 | 0 |
| 2 | Neg | Neg | Neg | Pf/ Pv | 0.4 | 0.7 |
| 4 | Pv | Pv | Pv | Pf/ Pv | 0.3 | 267.3 |
| 5 | Pv | Pv | Pv | Pf/ Pv | 0.2 | 10,830 |
| 3 | Neg | Neg | Pf | Pf/ Pv | 2.1 | 0.8 |
| 6 | Pf | Pf | Pf | Pf/ Pv | 124.3 | 0.6 |
| 7 | Pf | Pf | Pf | Pf/ Pv | 60.3 | 21.6 |
| 8 | Pf | Pf | Pf | Pf/ Pv | 555.6 | 0.6 |
| 9 | Pf | Pf | Pf | Pf/ Pv | 5,201 | 0.5 |
| 10 | Pf | Pf | Pf | Pf/ Pv | 7,215 | 0.3 |
| 11 | Neg | Neg | Neg | Pv | 0 | 0.4 |
| 12 | Neg | Neg | Neg | Pv | 0 | 0.6 |
| 13 | Neg | Neg | Pv | Pv | 0 | 4.0 |
| 14 | Neg | Pv | Neg | Pv | 0 | 1.57 |
| 15 | Neg | Neg | Neg | Pv | 0 | 0.5 |
| 16 | Neg | Neg | Neg | Pv | 0 | 0.2 |
| 17 | Neg | Neg | Neg | Pv | 0 | 0.6 |
| 18 | Neg | Neg | Neg | Pv | 0 | 0.4 |
| 19 | Neg | Neg | Neg | Pv | 0 | 0.9 |

Pf: *P. falciparum*, Pv: *P. vivax*, Neg: Negative
