# Supplementary material for: Validation study of Boil & Spin Malachite Green Loop Mediated Isothermal Amplification (B&S MG-LAMP) versus microscopy for malaria detection in the Peruvian Amazon
Source: PLoS One. 2021 Oct 25;16(10):e0258722. doi: 10.1371/journal.pone.0258722 (PMC8544869; doi:10.1371/journal.pone.0258722)
Supplement: Attachment — Submitted filename: PLOSone _Comments to the author.pdf [file pone.0258722.s007.pdf]

August 16th, 2021
Academic Editor
PLOS ONE

Dear Dr. Carvalho,

We appreciate your support and contributions as well as those of the reviewers to improve the quality of our manuscript entitled “Validation study of Boil & Spin Malachite Green Loop Mediated Isothermal Amplification (B&S MG-LAMP) versus microscopy for malaria detection in the Peruvian Amazon”.

Kindly find a point-by-point response to the reviewers’ concerns attached. We hope that our responses will be satisfactory and the manuscript will be accepted for publication.

Sincerely,
Hugo Valdivia, PhD
Head, Epidemiology Unit
Department of Parasitology
U.S Naval Medical Research Unit 6
Lima – Peru

RESPONSES

Comments to the Author

- Have the authors made all data underlying the findings in their manuscript fully available?

  Reviewer #1: No
  Response: We do not have any restrictions to provide an unidentified database. In this regard, we are including the individual tests results from the passive surveillance component on S2 table. Regarding the active surveillance database, individual results for the positive samples are described in Table 2. The remaining 493 samples of active surveillance were negative and we do not consider necessary to detail them in another supplementary table.

REVIEWER #1:

Major comments

1. In the "Methods" section, authors need to improve writing regarding the study design. As this is an accuracy study, I suggest that the authors follow the recommendations of the STARD 2015 E QUADAS-2 tool. Including a flowchart with the study design may help. In addition, it is recommended that authors add a sample calculation. Considering the information present in line 144 ("The samples tested in this study were selected from three protocols"), this study was carried out using panel samples from 3 different studies. It is necessary to know the number of samples for each of the studies, as well as the definition of their status (cases or non-cases based on the reference test result).

   Response: We appreciate the reviewer's suggestions. In this regard, we are providing a flowchart describing the process for the passive surveillance component on S1 fig. In addition, we included a paragraph regarding sample size calculation on the methods section. Regarding the studies and their status, the data is provided on Table 2 and S2 table. In summary, we have 258 (174 cases and 84 non-cases) from the first passive study, 25 subjects (4 cases and 21 non-cases) from the second passive study and 500 subjects (1 case and 499 non-cases) from the active surveillance.

2. They must also inform the average time between collection and extraction of DNA from these samples. Apparently, the inclusion of patients was not blinded or consecutive and microscopy was used as index test and as standard reference. This can also introduce bias and may have consequences in data interpretation. I suggest a paragraph disclosing the main limitations of the present article according to the STARD 2015 E QUADAS-2 tool.

   Response: The average time between sample collection and DNA extraction for the passive surveillance studies were 5 months. On the other hand, the average time between collection and DNA extraction for the active surveillance study was 2 months.

We used the STARD 2015 E and QUADAS-2 tools and found that there was a low bias in three areas.

For the passive surveillance study, i) samples were randomly selected, ii) the staff that performed subject enrolment was different from the one performing microscopy, iii) B&S MG-LAMP was performed in batches of 30 at the end of the week and iv) molecular technicians were blinded to the individual study files.

For the active surveillance study, i) enrolment staff were different from the laboratory staff, ii) the technician doing expert microscopy was different from the one running the B&S MG-LAMP in most cases and iii) B&S MG-LAMP was performed at least one month after microscopy, iv) samples were processed in batches of 30-60 and the technician did not have access to the subject study files making very hard to remember microscopy results.

A text referring the use of QUADAS is currently available in the methods section and on the discussion.

3. Another critical point is that with the data presented by the authors, it is not possible to calculate all the values presented in the topic "Diagnostic performance of microscopy and B&S MG-LAMP". The results must be clarified by showing in the contingency table used for calculation of the statistical parameters. I also suggest including a supplementary table with all the results for each of the samples. Another suggestion is to perform the accuracy analysis including the PET-PCR test as a reference standard.

   Response: Thank you for the suggestion. We are including S2 table with the individual results from the passive surveillance studies. For the active surveillance study, the results are presented in Table 2. We are not showing results from the remaining 493 samples since all of them were negative. Finally, table 1 now shows the results for all the laboratory tests. In this regard, calculations can be replicated using the information provided in any of both tables.

4. Another suggestion is to perform the accuracy analysis including the PET-PCR test as a reference

   Response: We appreciate the suggestion. However, the aim of the current work was to present the scientific community with a B&S MG-LAMP field deployable test and therefore it should be compared with other field deployable tests.

   It is important to notice that we are planning to test the modified B&S MG-LAMP employed in the active study using PET PCR and nested PCR as reference. However, this work is still ongoing and samples are still pending to be collected.

Minor comments

5. Line 247- include reference regarding the Cohen's Kappa coefficient.

   Response: Reference was included following your suggestion.

6. Lines 278-79 - In the text, the authors reported that B&S MG-LAMP detected 180 positive samples, but in Table 1 there are 179 (17 P. falciparum and 162 P. vivax).

   Response: Thank you for the suggestion; we have updated table 1 and the text on this section for accuracy.

7. "Line 292 :The species-specific B&S MG-LAMP identified 179 positives (17 P. falciparum, 162 P. vivax) , the genus B&S MG-LAMP identified 1 Plasmodium spp and 103 negatives."

   Response: Thank you for the suggestion; we have corrected the information for clarity.

8. Lines 320-21 - "Eight samples diagnosed as P. vivax by the PET-PCR were found to be negative by the B&S MG-LAMP". Include in the "Discussion", the impact that false-negative LAMP results may have on malaria control programs.

   Response: Thank you for the suggestion; we have included a paragraph in the discussion section.

9. Table 3-indicate that the cost calculation was estimated for 8 samples.

   Response: Thank you for the suggestion, the title in Table 3 was updated indicating in the equivalence "8 samples = 1 strip of tubes".

REVIEWER #2:

Major comments

1. The authors claim that increasing the amount of target DNA in the reaction tube by a factor of 1.6 (ie 10 uL of sample per 25 uL reaction instead of 5 uL sample per 20 uL reaction as used in the simplified protocol) improves assay performance in cases of low density parasitaemia. However, no data is presented comparing the simplified and modified protocols within the same study population, say during the active surveillance study, to allow for assessment of cost-effectiveness of the modified protocol.

   Response: Thank you for the insight provided for this manuscript. Regarding the modified B&S LAMP, we run a standard curve to document the limit of detection (LOD) and the estimated LOD was 0.5 parasites/µL for the genus and species-specific assays. Therefore, this result supports that this slight modification in master mix volumes and target DNA could potentially improve the assay performance for low parasitemia detection compared

to the simplified protocol. However, we are aware that a more rigorous evaluation of this modified mixed is required and we are stating that in the discussion section of manuscript.

In this regard, the comparison of the original MG-LAMP master mix versus the new master mix with increased target DNA that you kindly suggested goes beyond the scope of this manuscript. Furthermore, a bigger sample size of low parasitemia/submicroscopic cases is needed for an accurate comparison both methods. We are aiming to obtain sufficient samples from upcoming follow-ups of the active cohort study.

Minor comments

2. Title: replace "malaria detection"with "malaria diagnosis"

   Response: This change was performed as suggested.

3. L38-39: Replace "detect low-parasite densities" with "detect low-density parasitaemia"

   Response: Thank you for the suggestion, this change was updated as suggested.

4. in L232 it is stated that tenfold dilutions were done starting from 100 000 parasites/uL whereas in L262 and Fig 2 the starting parasite density is 10 000 p/uL

   Response: Thank you for finding the error, we have corrected it to 10,000 p/uL.

5. L256: replace ".....LoD...was one parasite/uL" with "...LoD was one parasite/uL or lower". The exact LoD is not known given that 1p/uL corresponds to the lowest density tested.

   Response: This change was corrected as suggested.

6. L322: Replace "seen" with "see"

   Response: Corrected as suggested.

7. Cost per reaction is stated as $1.2 in L408 and $9.60 in Table 3

   Response: This is because 9.6 showed in table 3 is the cost per a strip of 8 samples. We have added a text in the legend for clarification.
